# Single-port robotic non-transecting Y-V flap pyeloplasty with stricturoplasty for ureteropelvic junction obstruction: a case series

**DOI:** 10.1007/s00345-026-06551-6

**Published:** 2026-06-16

**Authors:** Wattanachai Ratanapornsompong, Sutthirat Sarawong, Aleksandra Walasek, Jeffery S. Lin, Amr Elbakry, Lee C. Zhao

**Affiliations:** 1 Department of Urology, NYU Langone Health, 11th Floor, 222 East 41st Street, New York, NY 10017 USA; 2 Division of Urology, Department of Surgery, Faculty of Medicine Ramathibodi Hospital, Mahidol University, Bangkok, 10400 Thailand; 3 Division of Urology, Department of Surgery, Police General Hospital, Bangkok, 10330 Thailand

**Keywords:** Ureteropelvic junction obstruction, Robotic pyeloplasty, Non-transecting reconstruction, Y–V flap, Single-port surgery

## Abstract

**Purpose:**

To report perioperative and short-term outcomes of single-port (SP) robotic vascular-preserving (non-transecting) Y-V flap pyeloplasty with stricturoplasty for ureteropelvic junction obstruction (UPJO). The most commonly performed pyeloplasty technique is the dismembered Anderson–Hynes technique; however, transection of the ureter compromises its blood supply and makes revision surgery more complex. Non-transecting pyeloplasty preserves the longitudinal ureteral blood supply and serves as an alternative to transecting techniques.

**Methods:**

All patients who underwent SP robotic-assisted non-transecting Y–V flap pyeloplasty with stricturoplasty for primary UPJO between December 2020 and December 2024 were retrospectively reviewed. Surgical success was defined as the absence of secondary intervention (endoscopic or surgical), resolution or improvement of symptoms, and stable or improved postoperative imaging findings.

**Results:**

A total of 21 cases met the inclusion criteria. Median operative time was 109 min. Median follow-up was 73 weeks. Overall surgical success was achieved in 95% of patients. There were no intraoperative complications, and the median estimated blood loss was 15 mL. Most patients were discharged the same day (18/21, 86%), with no Clavien–Dindo grade ≥ III complications. Postoperative renal scintigraphy was obtained in 8 patients, all of whom demonstrated improved drainage (T½ <20 min). The remaining patients were followed with clinical assessment and postoperative imaging. One patient developed recurrent obstruction after stent removal and was successfully managed with secondary endopyelotomy.

**Conclusions:**

SP robotic non-transecting Y-V flap pyeloplasty with stricturoplasty appears feasible and safe in selected patients with primary UPJO, with favorable short-term outcomes in this single-surgeon retrospective series. Further study with standardized functional follow-up is needed to define durability and appropriate patient selection.

**Supplementary Information:**

The online version contains supplementary material available at 10.1007/s00345-026-06551-6.

## Purpose

Multiple surgical techniques have been described for ureteropelvic junction obstruction (UPJO), with the dismembered Anderson–Hynes technique remaining the most commonly performed. It effectively addresses crossing vessels and high ureteral insertion, allows simultaneous pelvic reduction [1], and achieves high success rates. However, transection of the ureter may disrupt its longitudinal blood supply and increase the complexity of secondary reconstruction in cases of failure, which may require flaps or grafts [2, 3].

In urethral stricture surgery, non-transecting approaches have gained popularity to preserve vascular integrity [4]. We aimed to apply this principle to the upper urinary tract. Non-transecting pyeloplasty preserves ureteral blood supply, which may reduce ischemic complications and maintain dismembered pyeloplasty as a future option if revision is required. A potential limitation of flap pyeloplasty is a narrow ureteral plate at the UPJO, which may compromise reconstruction. To address this, we perform posterior ureteral stricturoplasty prior to flap advancement to widen the ureteral plate. Recent studies have shown that non-transecting pyeloplasty achieves outcomes comparable to dismembered pyeloplasty [1, 5–7].

Advances in single-port (SP) robotic systems have further facilitated non-dismembered reconstruction [8–10]. The supine anterior retroperitoneal access (SARA) approach further enhances the advantages of the SP platform [11]. We therefore report our experience with single-port robotic non-transecting Y–V flap pyeloplasty with stricturoplasty for primary UPJO.

## Methods

All patients who underwent SP robotic-assisted non-transecting Y–V flap pyeloplasty with stricturoplasty for primary UPJO between December 2020 and December 2024 were retrospectively reviewed from a prospectively maintained, institutional review board–approved database. The study was conducted in accordance with the Declaration of Helsinki. All procedures were performed using the da Vinci SP robotic system (Intuitive Surgical Inc., Sunnyvale, CA). All procedures were performed by a single surgeon, who adopted the non-transecting Y–V flap technique for cases with a non-obliterative UPJ. A “patent lumen” was defined as a discernible UPJ lumen that could be identified on preoperative imaging and confirmed intraoperatively by retrograde guidewire or flexible ureteroscopic assessment, indicating that the narrowed segment was amenable to a non-transecting reconstruction. Patients with obliterated UPJ segments not suitable for a non-transecting repair and those requiring multilevel ureteral reconstruction were excluded.

Baseline patient characteristics, perioperative variables, and postoperative outcomes were collected and analyzed. Baseline characteristics included age, sex, body mass index, laterality, and UPJO etiology. Perioperative variables included operative time, estimated blood loss, length of hospital stay, complications graded according to the Clavien–Dindo classification, and follow-up duration.

Surgical success was defined as a composite outcome including absence of secondary intervention (endoscopic or surgical), resolution or improvement of presenting symptoms, and stable or improved postoperative imaging findings. For patients who were asymptomatic at presentation, postoperative outcome was assessed by freedom from secondary intervention and stable or improved imaging findings rather than symptom response.

Preoperative functional assessment using renal scintigraphy, including differential renal function and drainage parameters, was recorded where available. Due to the retrospective nature of the study, these data were not uniformly available across all patients. Additional anatomical parameters, such as stricture length and pelvic configuration, were not consistently documented in the medical records and were therefore not included in the analysis.

### Operative technique

After induction of general anesthesia, the patient was placed in a supine (male) or lithotomy (female) position for urethral access.

A 3 cm incision was made at the point two-thirds between the anterior superior iliac spine and the umbilicus. This was carried down through the subcutaneous tissue to the fascia. Two stay sutures of 0 Vicryl were placed, the fascia was opened sharply, and the abdominal oblique muscles were spread bluntly to reveal the retroperitoneal space. A small incision was made, and the SP Access Port kit™ (Intuitive Surgical, Sunnyvale, CA) was placed. Pneumoretroperitoneum was established at 8 mmHg using an 8 mm AirSeal™ system (ConMed, Utica, NY) through the side port of the SP access port. Concomitant flexible cystoscopy was performed, and the ureter was cannulated with a guidewire. A flexible ureteroscope was advanced to the level of the obstruction.

**Fig. 1:**
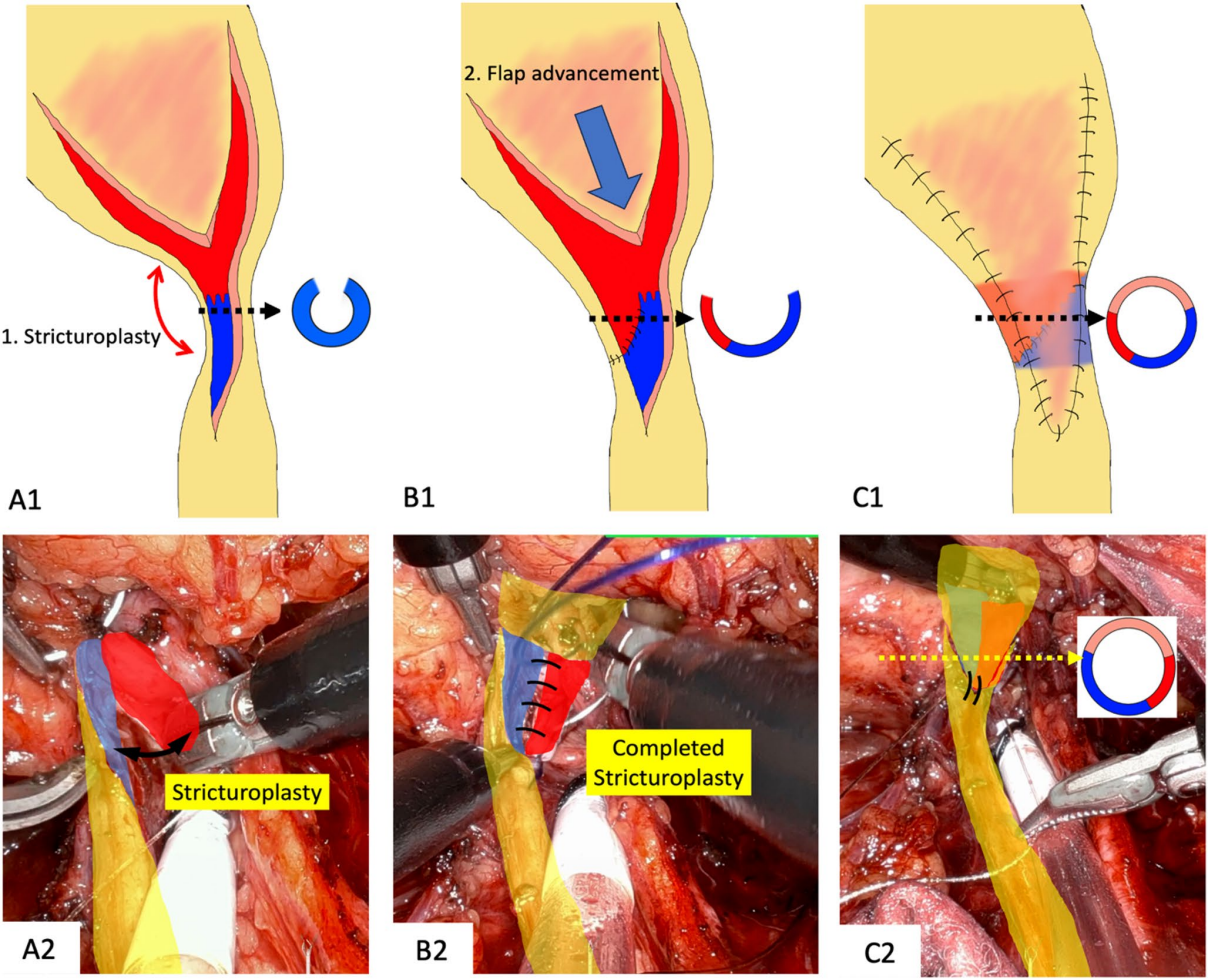
**A1**, **A2** Right ureteropelvic junction before augmentation with the flap. **B1** After stricturoplasty, the repair can be performed on either the medial or lateral wall, depending on which wall is more mobile. **B2** Demonstration of stricturoplasty performed on the medial side. **C1**, **C2** Ureteropelvic junction after flap augmentation. * Red indicates the posterior wall of the renal pelvis, blue indicates the ureter at the stricture site, and pink (flesh color) indicates the advanced flap used to cover the stricture.

Once the robot was docked, we dissected the retroperitoneum and identified the psoas major muscle. We followed the psoas muscle cranially as we lifted the retroperitoneal fat. We used near-infrared fluorescence (NIRF) in combination with light from a flexible ureteroscope to aid in identifying the UPJO. The ureter was traced cranially to the ureteropelvic junction at the level of the obstruction. A Y-shaped incision was made on the inferolateral renal pelvis and the lateral ureter, with the inferior limb of the Y extending through the UPJ into the proximal ureter. A renal pelvis flap was constructed to ensure a tension-free anastomosis to the apex of the spatulation. Renal pelvis mobilization was performed to increase flap mobility. A Y-to-V advancement was performed and anastomosed in a running fashion using a double-armed 4 − 0 barbed polydioxanone suture. Frequently, the ureteral plate at the UPJ was narrow, and we performed a stricturoplasty of the posterior ureteral wall prior to advancement of the renal pelvic flap, as shown in Fig. 1. A large renal pelvis can be reduced by excising the lateral renal pelvic tissue outside the V flap margins, as shown in Supplementary Fig. 1. The repair was completed over a ureteral stent that was placed retrogradely.

In cases with crossing vessels, identification of the ureteropelvic junction (UPJ) and renal pelvis was facilitated using retrograde flexible ureteroscopy and NIRF imaging. This allowed precise delineation of the anatomy and careful dissection of the UPJ away from the crossing vessels. Gentle blunt and sharp dissection was performed to mobilize both the ureter and the crossing vessel while preserving vascular integrity. Moreover, a cottonoid was used to aid in atraumatic retraction and to maintain separation between the ureteropelvic junction and the vessel during reconstruction. Importantly, the Y–V flap advancement and posterior stricturoplasty allowed relocation of the neo-UPJ to a position away from the crossing vessel (Supplementary Fig. 2), thereby minimizing the risk of vascular compression or traction on the repair. A formal Hellström maneuver [12] was not routinely performed.

The anastomosis was tested for watertight closure by filling the bladder with the stent in place. If there was concern about leakage or anastomotic integrity, a Foley catheter and/or surgical drain was considered. However, drains were not routinely used in this series.

In cases with a watertight anastomosis, a retroperitoneal approach, and internal drainage with a ureteral stent, additional drainage was not required. Any minor leak, if present, was expected to remain localized and could be managed with the stent alone. This approach also facilitated early mobilization and same-day discharge.

Supplementary videos are available in the online version of this article.

## Follow up

Most patients were discharged on the same day of the operation. If a Foley catheter was placed, it was removed on the same day prior to discharge. No drains were used in this series. Ureteral stents were removed approximately 2–3 weeks postoperatively using office flexible cystoscopy under local anesthesia. All patients underwent routine postoperative imaging with renal ultrasound or contrast-enhanced CT at approximately 4 weeks after stent removal, at 6, and 12 months, and then annually. In patients with symptomatic improvement and reduced hydronephrosis, no further functional imaging was performed. If symptoms persisted or imaging findings were unclear, additional evaluation with diuretic renography was obtained. High-grade obstruction was defined as T½ >20 min. In cases with evidence of recurrent obstruction, endoscopic intervention was pursued.

## Results

A total of 21 patients were included between December 2020 and December 2024. The median age was 35 years, and 16 patients (76%) were female. Flank pain was the most common presenting symptom (14/21, 69%). Three patients presented with urinary tract infection, and four were asymptomatic with incidentally detected UPJO. Etiologies included congenital obstruction (9/21), crossing vessels (5/21), high ureteral insertion (5/21) (Two patients had both crossing vessels and high ureteral insertion), idiopathic UPJO (3/21), and one iatrogenic case. Two patients (9.5%) had undergone prior endopyelotomy before surgical reconstruction.

Preoperative drainage parameters (T½) were available in 15 of 21 patients. Among these, measurable T½ values were recorded in 6 patients (median 17.8 min, IQR 15–28), while drainage was not achieved in 9 patients. Preoperative differential renal function of the affected kidney was available in 16 of 21 patients, with a median value of 43.5% (IQR 34.5–48).

Baseline characteristics are summarized in Table 1.

There were no intraoperative complications or conversions. The median estimated blood loss was 15 mL. An assistant port was used in the first three cases early in the series.

Eighteen patients (86%) were discharged on the day of surgery, with a median length of stay of 0 days (range 0–1). Three patients were admitted overnight. A Foley catheter was used in five cases (24%) and removed on the same day. In all cases, intraoperative assessment confirmed a watertight anastomosis. Foley catheter placement was not inserted due to concern for anastomotic integrity, but was requested by the anesthesia team for postoperative urine output monitoring. No significant urinary spillage was observed, and no surgical drains were used. No Clavien–Dindo grade ≥ III complications occurred during the perioperative or follow-up period.

Median follow-up was 73 weeks, with no loss to follow-up. Surgical success was achieved in 20 of 21 patients (95.2%). All ureteral stents were removed as planned at 2–3 weeks. Among the 8 patients who underwent postoperative renal scintigraphy, all demonstrated a T½ < 20 min. Most (7/8) had T½ <10 min, except for one patient (15.2 min), who improved from a preoperative non-draining system, with resolution of symptoms and improvement in hydronephrosis. Of the remaining 13 patients, 12 were followed with clinical assessment and imaging, demonstrating improvement in hydronephrosis and resolution of symptoms. One patient developed recurrent flank pain following stent removal and was found to have persistent obstruction. This patient underwent secondary endopyelotomy and subsequently achieved clinical and radiographic improvement.

**Table 1:** Baseline, perioperative, and postoperative outcomes of patients undergoing single-port robotic non-transecting Y–V flap pyeloplasty

Total	21
Female, n (%)	16 (76%)
Median Age (years), IQR	35 (25–57)
Median body mass index (IQR), kg/m2	24.6 (20.5–29.7)
Laterality, n (%)	
Left	5 (23.8)
Right	16 (76.2)
Etiology of UPJO, n (%)	
Congenital	9 (39)
Idiopathic	3 (13)
Crossing vessel*	5 (22)
High insertion*	5 (22)
Iatrogenic	1 (4)
Prior intervention (endopyelotomy), n (%)	2 (10)
Operative time (min), median (IQR)	109 (90–143)
Estimated blood loss (mL), median (IQR)	15 (5–50)
Length of stay (days), median (range)	0 (0–1)
Postoperative complications, Clavien–Dindo grade ≥ III within 30 days, n	0
Follow-up (weeks), median (IQR)	73 (24–127)
Surgical success (%)	20/21 (95.2)

## Discussion

In this selected single-surgeon series, SP robotic non-transecting Y–V flap pyeloplasty with stricturoplasty was associated with low perioperative morbidity, a high rate of same-day discharge, and favorable short-term outcomes. Freedom from secondary intervention was observed in 20 of 21 patients (95%), consistent with previously reported series [3, 13]. Supplementary Table 1 summarizes the reported outcomes of non-dismembered flap pyeloplasty in the literature.

Historically, the Anderson–Hynes pyeloplasty has been considered the gold standard due to its high success rates and its ability to address a broad range of etiologies, including high ureteral insertion, crossing vessels, and aperistaltic segments. However, several studies have reported that non-dismembered flap pyeloplasty can achieve outcomes comparable to dismembered pyeloplasty [14], with potential advantages such as technical simplicity, fewer sutures, and shorter operative time [15]. From a physiological perspective, preservation of the ureteral plate may reduce the risk of ischemic injury. Additionally, flap-based pyeloplasty may be beneficial in long-segment narrowing or a small renal pelvis [16, 17].

Several studies have compared non-dismembered flap pyeloplasty with the dismembered technique [1, 5, 18, 19] (Supplementary Table 1). However, two studies have reported lower efficacy for non-dismembered approaches. In pediatric patients, Casale et al. reported a 94% resolution rate with dismembered pyeloplasty compared with 43% with Heineke–Mikulicz flap repair. They attributed the lower success rate to retention of diseased fibrotic tissue in non-dismembered repair, potentially leading to impaired healing [20]. We believe that a narrow ureteral plate may be an additional factor contributing to failure, which may not be adequately addressed by the Heineke–Mikulicz technique. To address this, we perform posterior ureteral stricturoplasty to widen the ureteral plate and incorporate more healthy pelvic tissue into the anastomosis.

Klingler et al. reported a 73% success rate for Y–V pyeloplasty in a small series of 15 patients and suggested that this approach may be less effective in the setting of a significantly enlarged renal pelvis [21]. The necessity of routine pelvic reduction during pyeloplasty remains debated [1, 15]. In our Y–V flap technique, flap creation inherently reduces pelvic redundancy, and additional excision can be performed when indicated (Supplementary Fig. 1).

In cases of UPJO associated with crossing vessels, routine anterior transposition of the renal pelvis may not always be necessary [22, 23]. Given the anterior location of renal vessels and limitations of transposition, alternative strategies may be appropriate. The vascular hitch technique has demonstrated favorable outcomes in selected patients [24]. With the Y–V flap technique, the ureteropelvic junction is relocated inferiorly, potentially relieving vascular compression without formal transposition, as illustrated in Supplementary Fig. 2. If secondary endoscopic management is required, the incision should be planned with careful review of postoperative anatomy and the location of crossing vessels on imaging, avoiding the vascular side of the UPJ. In such cases, endopyelotomy must be individualized and performed cautiously because the presence of crossing vessels may limit safe incision orientation.

In our practice, dismembered (Anderson–Hynes) pyeloplasty is the preferred approach for obliterated or severely fibrotic UPJ segments where non-transecting reconstruction is not feasible, as well as for complex anatomy, such as malrotated or horseshoe kidneys. For longer obliterated segments that preclude a tension-free anastomosis, we adopt augmented techniques, including augmented anastomotic repair or buccal mucosa graft onlay ureteroplasty.

The robotic platform enhances visualization and suturing precision and may offer advantages in recovery and surgeon ergonomics [25]. NIRF, when combined with ureteroscopy, may help identify the stenotic segment. It can also be used to assess flap perfusion using intravenous indocyanine green. The SP platform enables a retroperitoneal approach in the supine or lithotomy position, facilitating patient positioning and concurrent ureteroscopy without repositioning [11, 26], which makes patient positioning easier and more favorable for anesthesia. Compared with multiport systems, the SP platform provides more direct access to the renal pelvis with less dissection. Although this technique can also be performed with a multiport system, the SP platform offers practical advantages in access and ergonomics.

## Limitations

This study has several limitations. It is a retrospective case series from a single institution and reflects the experience of a single high-volume reconstructive surgeon, which may limit generalizability. The sample size is relatively small, although this reflects both the selective use of this technique and the rarity of the procedure. There is no comparison group; therefore, no conclusions can be made regarding superiority or equivalence relative to dismembered pyeloplasty or other minimally invasive approaches. Patient selection was limited to cases with a patent lumen suitable for a non-transecting reconstruction, which introduces selection bias. Standardized preoperative variables, including drainage half-time, hydronephrosis severity, and stricture characteristics, were not consistently available, and postoperative renal scintigraphy was obtained selectively rather than routinely in all patients. Longer-term data are needed to define durability.

## Conclusion

Single-port robotic vascular-preserving (non-transecting) Y-V flap pyeloplasty with stricturoplasty for the treatment of ureteropelvic junction obstruction achieves success rates comparable to traditional techniques. This procedure can be performed through a retroperitoneal approach with a high rate of same-day discharge.

## Supplementary Information

Below is the link to the electronic supplementary material.

Supplementary Material 1

Supplementary Material 2

## Data Availability

The datasets generated and/or analyzed during the current study are not publicly available due to institutional data protection regulations but are available from the corresponding author on reasonable request.
