# Supplementary material for: Single-port robotic non-transecting Y-V flap pyeloplasty with stricturoplasty for ureteropelvic junction obstruction: a case series
Source: World J Urol. 2026 Jun 16;44(1):433. doi: 10.1007/s00345-026-06551-6 (PMC13272238; doi:10.1007/s00345-026-06551-6)
Supplement: Supplementary file 2 — Supplementary Material 2 [file 345_2026_6551_MOESM2_ESM.docx]

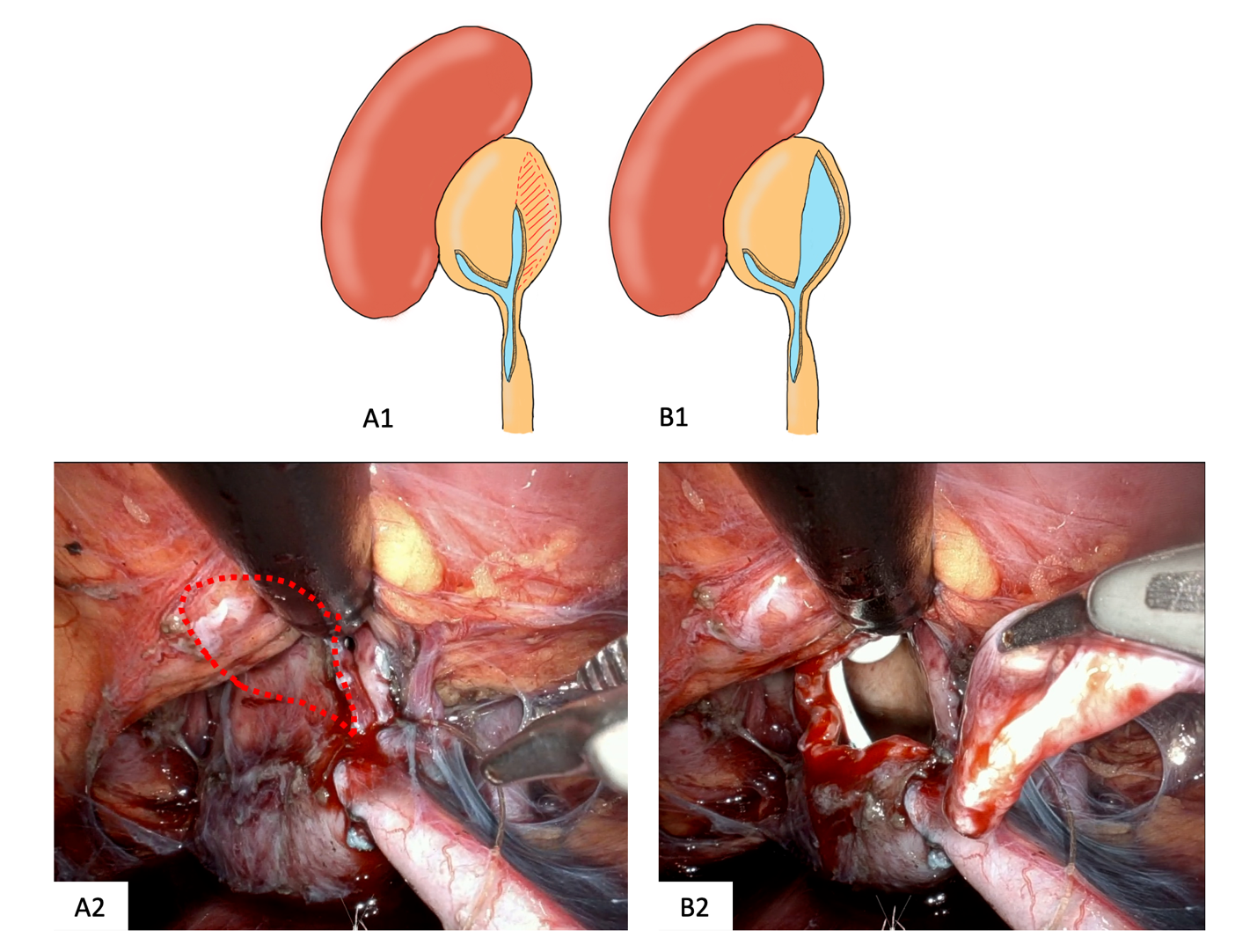


Supplementary Figure 1. Shows the pelvic reduction technique in right-sided pyeloplasty.

**
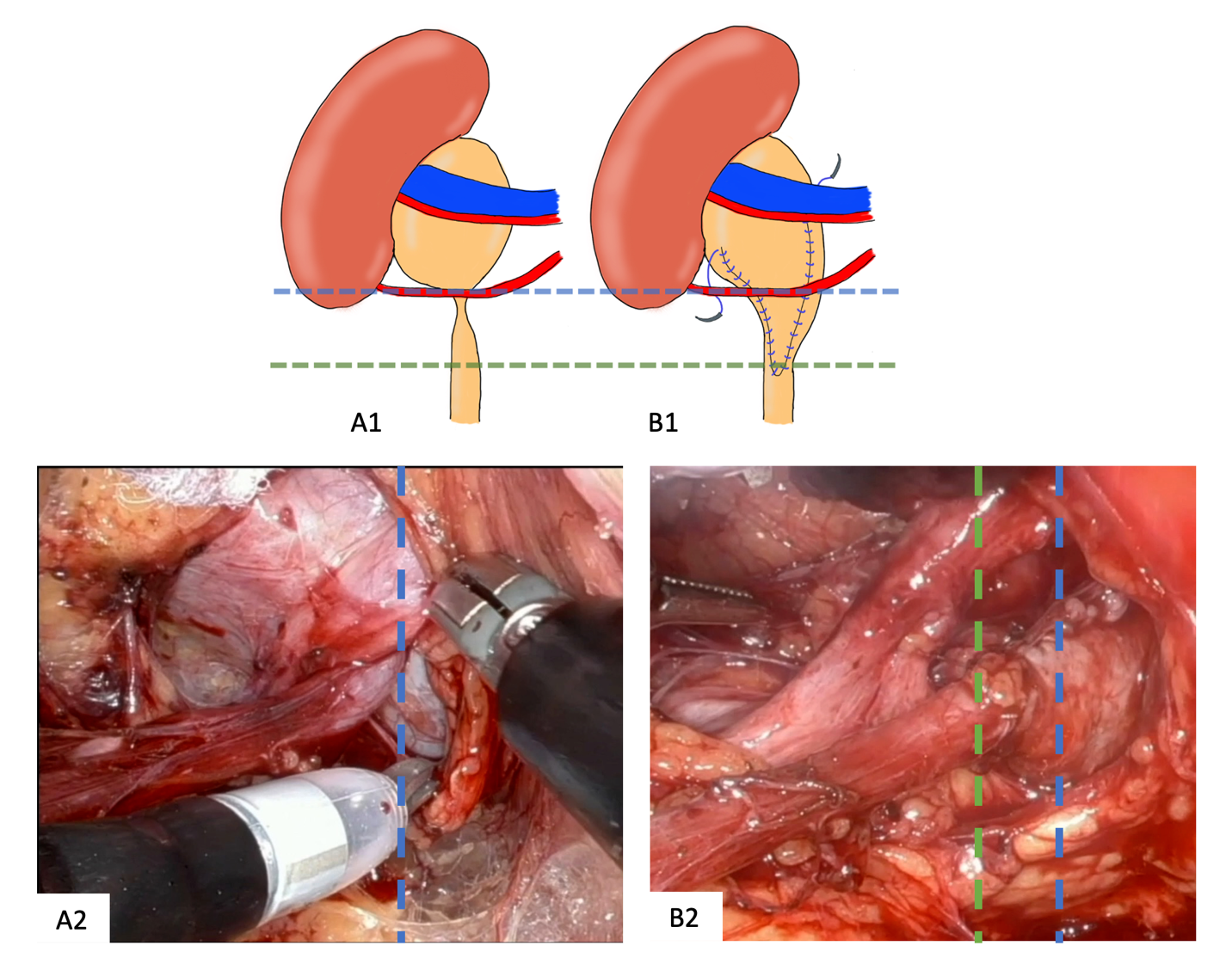
**

Supplementary Figure 2. Illustration showing how the new ureteropelvic junction (UPJ) is moved away from the crossing vessel after left-sided pyeloplasty. The blue line indicates the original UPJ position, and the green line indicates the new UPJ position after pyeloplasty.

# Supplementary Table 1. Summary of Published Studies on Minimally Invasive Non-transecting Pyeloplasty

| Study (Author, Year) | Single vs. Multi-Institution | No. of Patients | Type of Repair | Definition of Success | Success Rate (%) |
| --- | --- | --- | --- | --- | --- |
| Klingler et al., 2003 | Single institution | 15 | Laparoscopic Pyeloplasty (Y-V: 5, Fenger: 10) | Absence of symptoms + reduction of hydronephrosis on US/IVP + no obstruction on IVP/Renal scan | 73.3% (11/15) |
| Casale et al., 2004 | Multi-institution | 7 | Laparoscopic pyeloplasty (Heineke–Mikulicz, HM) | Resolution of UPJ obstruction based on symptoms and postoperative imaging (ultrasonography and/or diuretic radionuclide scan) | 43% |
| Rassweiler et al., 2007 | Single institution | 68 | Retroperitoneoscopic pyeloplasty (Y-V) | >80% pain relief (symptomatic resolution) AND stable or improved renal function AND improved washout (T½ < 20 min on renal scan / excretory urography) AND resistance index (RI) < 0.7. | 97.1% |
| Subotic et al., 2013 | Multi-institution | 21 | Retroperitoneoscopic pyeloplasty (Y–V) | Symptomatic resolution (>80% pain relief) plus stable or improved renal function, improved washout (T½ < 20 min) on renal scan / excretory urography, and resistance index (RI) < 0.75 | 90.5% |
| Nerli et al., 2014 | Single institution | 11 | laparoscopic Foley’s YV plasty | Improved drainage and renal function on renogram at 3 months; post-op | 100% |
| Haga et al., 2015 | Multi-institution | 26 | Laparoscopic modified bypass pyeloplasty (non-dismembered side-to-side ureteropelvic anastomosis) | Marked reduction in hydronephrosis on ultrasound, preservation of split renal function, improved drainage curve on diuretic renography, and symptom resolution at 6 months | 96% |
| Cheng et al., 2021 | Single institution | 21 | Modified minimally invasive flap pyeloplasty (laparoscopic + robotic) using “wishbone” anastomosis ± “ureteral plate” | Alleviation of subjective symptoms (flank pain) and improvement of hydronephrosis | 90.5% |
| Jarrett et al., 2002 | Single institution | 28 | Y-V plasty + Heineke–Mikulicz | Freedom from obstruction on follow-up radiographic imaging ± symptom resolution | 96% overall (not technique-specific) |
| Szydełko et al., 2010 | Single institution | 36 | Laparoscopic Y–V pyeloplasty | Absence of any clinical symptoms plus significant reduction of hydronephrosis on IVU & ultrasound, and no sign of obstruction on IVU and/or diuretic renography (T½ < 12 min). | 91.2% |
| Rassweiler et al., 2018 | Single institution | 131 | Retroperitoneal laparoscopic YV-pyeloplasty (LRYVP) for UPJO with crossing vessels | “Overall success”: improvement of drainage on IVP/diuretic renogram or improvement of hydronephrosis on ultrasound, with no deterioration of renal function | 90% (118/131) |
